# Magnetoresistance and Magnetic Relaxation of La-Sr-Mn-O Films Grown on Si/SiO_2_ Substrate by Pulsed Injection MOCVD

**DOI:** 10.3390/s23125365

**Published:** 2023-06-06

**Authors:** Nerija Žurauskienė, Vakaris Rudokas, Sonata Tolvaišienė

**Affiliations:** 1 Department of Functional Materials and Electronics, Center for Physical Sciences and Technology, Sauletekio Ave. 3, LT-10257 Vilnius, Lithuania; vakaris.rudokas@ftmc.lt; 2 Faculty of Electronics, Vilnius Gediminas Technical University, LT-10223 Vilnius, Lithuania; sonata.tolvaisiene@vilniustech.lt

**Keywords:** colossal magnetoresistance, MOCVD technology, nanostructured manganite films, resistance-relaxation processes, pulsed magnetic field, magnetic-field sensors, Si/SiO_2_ substrate

## Abstract

The results of magnetoresistance (*MR*) and resistance relaxation of nanostructured La_1−x_Sr_x_Mn_y_O_3_ (LSMO) films with different film thicknesses (60–480 nm) grown on Si/SiO_2_ substrate by the pulsed-injection MOCVD technique are presented and compared with the reference manganite LSMO/Al_2_O_3_ films of the same thickness. The *MR* was investigated in permanent (up to 0.7 T) and pulsed (up to 10 T) magnetic fields in the temperature range of 80–300 K, and the resistance-relaxation processes were studied after the switch-off of the magnetic pulse with an amplitude of 10 T and a duration of 200 μs. It was found that the high-field *MR* values were comparable for all investigated films (~−40% at 10 T), whereas the memory effects differed depending on the film thickness and substrate used for the deposition. It was demonstrated that resistance relaxation to the initial state after removal of the magnetic field occurred in two time scales: fast’ (~300 μs) and slow (longer than 10 ms). The observed fast relaxation process was analyzed using the Kolmogorov–Avrami–Fatuzzo model, taking into account the reorientation of magnetic domains into their equilibrium state. The smallest remnant resistivity values were found for the LSMO films grown on SiO_2_/Si substrate in comparison to the LSMO/Al_2_O_3_ films. The testing of the LSMO/SiO_2_/Si-based magnetic sensors in an alternating magnetic field with a half-period of 22 μs demonstrated that these films could be used for the development of fast magnetic sensors operating at room temperature. For operation at cryogenic temperature, the LSMO/SiO_2_/Si films could be employed only for single-pulse measurements due to magnetic-memory effects.

## 1. Introduction

In recent years, the demand for magnetic sensors based on magnetoresistive (MR) technologies has rapidly increased, especially when enhanced sensitivity, detectivity, and miniaturization are required [1,2]. A recent report by the Yole Group on the magnetic-sensor market and technology [3] predicted an increase in the MR-sensor market at the expense of widely used Hall sensors in consumer, automotive, medical, industrial, and other application areas. Magnetoresistive sensors, as they are broadly understood, usually include xMR (anisotropic AMR, giant GMR, and tunneling TMR) devices, and their operation range in magnetic fields is less than tens of millitesla [4]. However, advanced scientific, medical, and industrial equipment requires magnetic-field sensors capable of measuring stronger magnetic fields (1–10 Tesla) in a wide range of temperatures [5]. Furthermore, the application of high-pulsed magnetic fields for scientific investigations or in industry requires fast, highly sensitive magnetic sensors with nano-microscale dimensions.

From this point of view, mixed-valence manganites with a perovskite structure have been widely studied as potential materials for magnetic sensors [2,6], magnetic refrigeration [7], data storage [8], and other multifunctional-device applications. Manganite perovskites (general chemical formula Re_1−x_A_x_MnO_3_, where Re is rare-earth cation, and A is alkali or alkaline-earth cation), depending on the chemical composition, show a rich variety of crystallographic, electronic, and magnetic phases. An interesting magnetic-field-induced phase diagram can be obtained for highly frustrated hexagonal manganites (for example, HoMnO_3_) [9], which is important for application in quantum-spin systems at low temperatures. For magnetic-field-sensor applications, the colossal-magnetoresistance (CMR) effect has been extensively studied in various manganite-oxide compounds. The CMR effect can be qualitatively explained by a double-exchange interaction between manganese ions Mn^4+^ and Mn^3+^. The application of an external magnetic field aligns the spins of the manganese ions and their itinerant electrons, facilitating the electron transport and leading to a significant decrease in resistivity. However, the double-exchange mechanism alone is not sufficient to fully explain the CMR effect. The Jahn–Teller effect, which causes the distortion of the crystal lattice around certain manganese ions and gives rise to a strong electron–phonon interaction, must also be taken into consideration [10]. Therefore, the interplay between magnetic and lattice properties results in the colossal change in manganite resistivity. It is worth mentioning that the highest magnetoresistance values in high-quality monocrystals or epitaxial films are observed only close to the Curie temperature, which limits their application. Consequently, many attempts were directed to the study of polycrystalline or nanostructured films, which demonstrate so-called extrinsic magnetotransport phenomena [11]. These films exhibit significant magnetoresistance in a broad temperature range, from phase-transition temperature down to cryogenic temperatures [12].

It was demonstrated that magnetic sensors based on the CMR effect in polycrystalline (nanostructured) manganite films are capable to measure pulsed-magnetic-field magnitude independently on magnetic-field orientation in respect to the sensor’s plane [13,14]. Such sensors, called CMR-B-scalar sensors [15], were used for the measurement of magnetic-field distribution in electromagnetic launchers and nondestructive pulsed magnets when the duration of magnetic-field pulses was more than one millisecond [14,16]. However, for shorter pulses, for example, in magnetic forming and welding systems or plasma-science applications, sensors measuring short (microsecond duration) magnetic fields with high accuracy and temporal resolution are required [17]. Therefore, to use manganite films for the development of fast magnetic sensors, the magnetic memory effects have to be minimized. The memory effects in manganites are related to the magnetization relaxation of the films upon the reversal or removal of an external magnetic field. Sirena et al. [18] demonstrated that magnetization-relaxation phenomena are interlinked with resistance relaxation and could be investigated by measuring the resistance dynamics after the removal of the magnetic field [18]. The decrease in magnetization implies a rise in magnetic disorder, leading to a corresponding increase in resistivity, and a relaxation to the initial value can be observed. Therefore, similar behavior has been found in both the magnetization- and resistivity-dynamic measurements [18]. Various mathematical expressions have been proposed to describe the observed dynamics of magnetization and resistance relaxation in different materials, including logarithmic, power–law, exponential (Debye), and compressed or stretched exponential decay, as cited in refs. [18,19,20,21,22,23,24]. It has been demonstrated that magnetic-relaxation processes are strongly influenced by the structural quality of the films, which is determined by various factors, including fabrication conditions, the substrates used, film thickness, chemical composition, etc.

To increase the complexity and functionality of MR sensors and possibility develop a sensor system on a chip, compatibility with semiconductor-based technologies is required [25,26]. Therefore, the development of growth technologies of magnetoresistive films on Si/SiO_2_ substrates compatible with the standard Si technology is of great importance [27]. Si/SiO_2_ substrates are relatively inexpensive compared to other substrates, such as NdGaO_3_ (NGO), LaAlO_3_ (LAO), or sapphire, which makes them a more cost-effective option for large-scale production. Moreover, Si/SiO_2_ substrates have good thermal stability, which allows for high-temperature processing and annealing of manganite thin films, leading to better crystallinity and improved properties. However, lattice mismatch with manganite films can lead to strain and the introduction of defects in the thin film, as well as the formation of an interfacial layer, which can affect the film properties [28,29]. Therefore, the investigation of magnetic and transport properties of lanthanum-manganite films grown on Si/SiO_2_ substrate is of great importance for the development of magnetic sensors and other spintronic devices.

It is worth mentioning that lanthanum manganites doped with a certain amount of Sr (*x*) exhibit relatively high Curie temperature *T*_C_ and metal-insulator transition temperature *T*_m_, which extends the operation temperature range of manganite-based devices up to room temperature. Although for good-quality epitaxial films the highest *T*_C_ and *T*_m_ values were obtained for *x* ≈ 0.33, the optimal composition for polycrystalline-nanostructured films was found when *x* ≈ (0.17–0.2) [30].

In this study, we present the results of the investigation of magnetoresistance and its relaxation of La_1−x_Sr_x_MnO_3_ (*x* ≈ 0.17) films grown on Si/SiO_2_ substrate by the pulsed-injection metal–organic chemical-vapor deposition (PI MOCVD) technique. The capability of measuring high-pulsed magnetic fields with short (microseconds) pulse duration at room and cryogenic temperatures are presented and discussed.

## 2. Materials and Methods

### 2.1. Film Preparation

A set of La_1−x_Sr_x_(Mn)_z_O_3_ films was deposited onto an Si/SiO_2_ substrate (SiO_2_ thickness ≈ 1000 nm) with varying film thicknesses of 60 nm, 160 nm, 360 nm, and 480 nm using the PI MOCVD technique. The thickness of the films was evaluated by the time of the deposition and determined more accurately using a profilometer. The growth rate of the films was ~28 nm/min. A polycrystalline Al_2_O_3_ substrate, which was used for the deposition of nanostructured manganite films in previous studies [30,31,32], was used as a reference for comparison of the magnetoresistive properties. The LSMO films of the same thickness were grown during the same deposition process on SiO_2_ and Al_2_O_3_ substrates. The required chemical composition was achieved by preparing an organic solution with a mixture of metal–organic precursors of La(thd)3, Sr(thd)2, Mn(thd)3 (thd is 2,2,6,6-tetramethyl-3,5-heptandionate) dissolved in monoglyme as the source. The microdoses of this organic solution were injected into the diffuser, which was heated to 300 °C during the growth process. The resulting vapor mixture was then transported by Ar + O_2_ (3:1) gas towards the substrate, which was heated to 750 °C. Once the growth process was completed, the film was post-annealed for 10 min in an oxygen atmosphere to ensure the required oxygen-saturation level in the grown films. More detailed information on a similar film preparation can be found in [31,33].

### 2.2. Characterization

**Figure 1:**
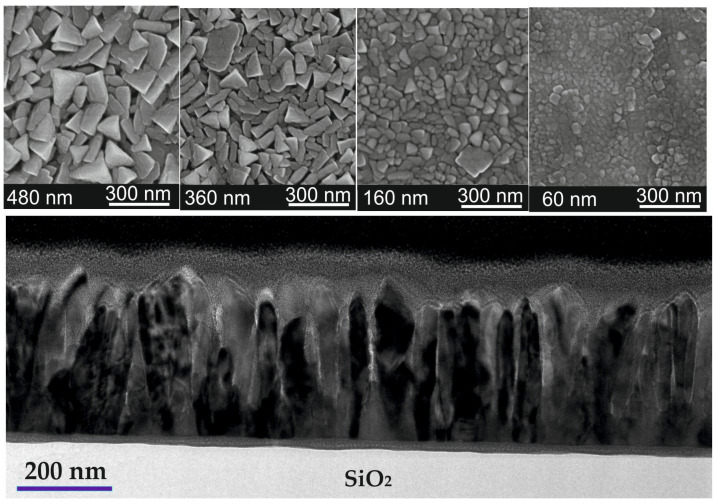
SEM surface images of La_0.83_Sr_0.17_Mn_1.21_O_3_ films with different thickness (**upper images**) and TEM image of the film with a thickness of 360 nm (**bottom image**).

The La_1−x_Sr_x_Mn_y_O_3_ (LSMO) films were analyzed using inductively coupled plasma high-resolution mass spectrometry (ICP-MS) to determine their elemental composition with a high accuracy of ±0.01. The final chemical composition of the films was La_0.83_Sr_0.17_Mn_1.21_O_3_, with a slightly decreased Sr content for 60 nm-tick films. The microstructure of the films was examined using transmission electron microscopy (TEM) to investigate their internal structure and scanning electron microscopy (SEM) to determine their surface morphology. The SEM images in Figure 1 reveal that the surface of all LSMO films grown on Si/SiO_2_ substrates consisted of crystallites with predominantly triangular shapes. As the film thickness increased, the crystallites became more visible and larger in size. For instance, in a 60 nm-thick film, the average size of the crystallites was about 40 nm, whereas in a 480 nm-thick film, they could reach up to 200 nm. In reality, the films consisted of separate crystallite columns that extended through the film thickness, as seen in the low-magnification cross-sectional TEM image in the bottom image of Figure 1. These columns were monocrystalline or composed of twinned monocrystals, with the long axis perpendicular to the substrate. The contrast of the TEM images shows an intermediate gray layer between the SiO_2_ substrate and the films, which was about 20 nm thick for the films with a thickness of 360 nm and decreased with the decrease in film thickness. Previous studies on similar films [34] have shown that this layer is the result of mutual diffusion of LSMO film elements into the substrate and vice versa. It should be noted that regardless of the amorphous surface of the SiO_2_ substrate, the films grew in the form of monocrystalline columns.

**Figure 2:**
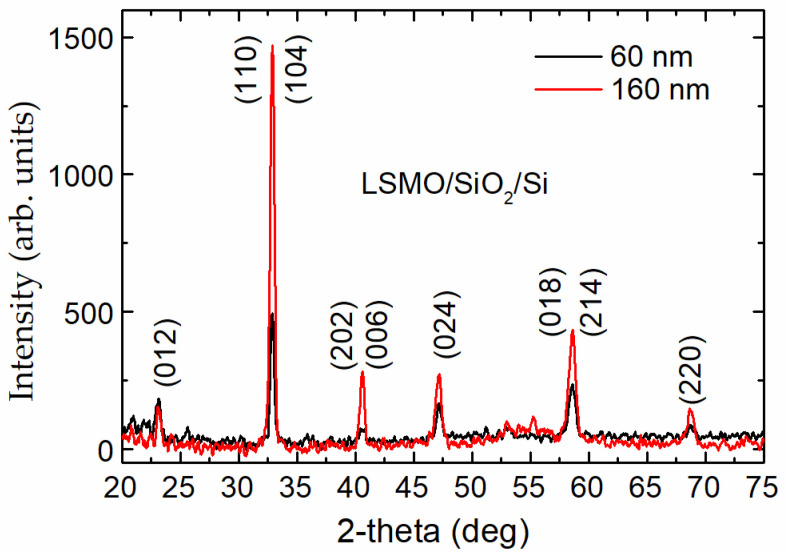
X-ray diffraction patterns in grazing-incidence geometry for two LSMO/SiO_2_/Si films with different thicknesses (60 nm and 160 nm). All peaks are assigned to the reflections from a rhombohedral perovskite lattice (indices are given for hexagonal unit cell).

Grazing-incidence X-ray-diffraction (GIXRD) measurements of the films were performed using a SmartLab diffractometer. The angle of the incident X-ray beam was fixed at 0.5°. Figure 2 shows the GIXRD spectra of two LSMO/SiO_2_/Si samples with different thicknesses. Only the characteristic peaks associated with the single-phase perovskite-like polycrystalline structure with rhombohedral distortions (the space group R3c) were observed. Moreover, increasing the thickness of the films led to the increase in peak intensity, without other observable changes. The X-ray diffraction of the films grown on Al_2_O_3_ also revealed a single-phase polycrystalline structure (see, for example, [31]).

To conduct electric-transport and magnetoresistance measurements, two Ag electrodes with a Cr sublayer were thermally deposited and then post-annealed for 1 h in an Ar atmosphere at 450 °C.

The dependence of the resistivity (*ρ*) on temperature was investigated within a temperature range of 80–300 K using a closed-cycle helium-gas cryocooler. Magnetoresistance (*MR*) was defined as *MR*(%) = 100 × [*ρ*(*B*)/*ρ*_0_ − 1], where *ρ*(*B*) and *ρ*_0_ represent the resistivity at magnetic-flux density *B* and without a magnetic field, respectively. The *MR* vs. *B* dependences were studied within a temperature range of 80–300 K using both an electromagnet with a permanent magnetic field of up to 0.7 T and a non-destructive pulsed magnet generating magnetic fields of up to 10 T through a multi-shot magnetic-field coil [35]. For the measurement of magnetic-relaxation processes following the switch-off of the magnetic-field pulse, a specially fabricated coil with a non-metallic outer casing made of polyamide was used to enable the rapid switch-off of the magnetic pulse [36]. This coil enabled half-sine-wave magnetic-field pulses with amplitudes of up to 10 T and durations of 200 μs to be produced. The response signal (*V*_res_) was measured by passing a current of 0.2 mA through the manganite sample and recording the resistance change across the sample due to change in magnetic field.

Furthermore, to explore the potential use of the grown films in the development of high-speed magnetic-field sensors capable of measuring short, microsecond-duration, pulsed magnetic fields, an alternating magnetic-field generator was employed. This generator was composed of a capacitor with a capacitance of 20 μF and a double-layer coil with an inductance of 1 μH. A high-voltage relay functioned as a switch. Upon discharge of the capacitor, an oscillating magnetic field with gradually diminishing amplitude was produced. By charging the capacitor to 4 kV, a maximum magnetic-flux density of about 3 T with a half-period duration of 22 µs was generated. In order to eliminate the unwanted voltage that is induced in the transmission line of a sensor due to electromotive forces during the measurement of the short-pulsed magnetic field, a measurement module using a high-frequency bipolar-pulsed voltage supply was employed for the testing of the CMR sensor [32].

## 3. Results and Discussion

### 3.1. Resistivity of LSMO/SiO_2_/Si Films: Comparison with LSMO/Al_2_O_3_ Films

**Figure 3:**
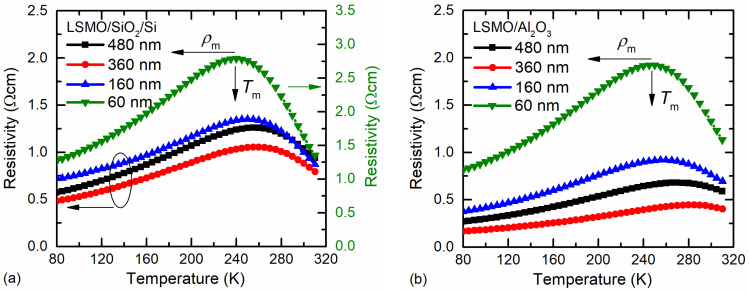
Resistivity vs. temperature dependences of LSMO films with different thicknesses grown on Si/SiO_2_ (**a**) and polycrystalline Al_2_O_3_ (**b**) substrates.

The resistivity vs. temperature dependences for LSMO films of different thicknesses are presented in Figure 3. The resistivity maximum (*ρ*_m_) of the thinnest films was the highest, whereas the insulator–metal-transition temperature *T*_m_ was the lowest. This can be explained by the more defective structure of the thin films. Whereas the XRD measurements revealed a single-phase structure of the films, the half-peak width was slightly wider for thinner films (see Figure 2), indicating smaller crystallites. The SEM images presented in Figure 1 clearly show that the thinnest films had the smallest crystallites; thus, the number of grain boundaries in the same volume of film was the largest in comparison with the thicker films, which resulted in higher resistivity. However, for the 480 nm-thick film, the *T*_m_ was shifted to lower temperatures and the *ρ*_m_ was increased in comparison to the 360 nm-thick film. This could be explained by the higher amount of disorder accumulated in the grain boundaries between column-shaped crystallites during the longer growth of thicker film. Moreover, higher resistivity values were obtained for films grown on the amorphous SiO_2_ substrate in comparison to the polycrystalline Al_2_O_3_ one. This is a result of a higher level of disorder in the film grown on the amorphous surface. Moreover, the interdiffusion layer that was observed close to the SiO_2_ substrate could also have had an influence on the more disordered films and imperfect columns of crystallites that were composed of several sub-columns (twinned monocrystals), as can be seen in the TEM image in Figure 1.

The main parameters of the LSMO films with different thicknesses are compared in Table 1.

### 3.2. Magnetoresistance of LSMO Films

**Figure 4:**
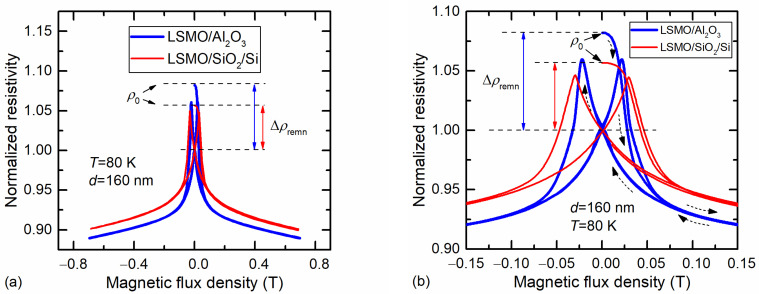
Normalized resistivity change on the magnetic field (three cycles) for LSMO films with a thickness of 160 nm grown on Si/SiO_2_ and Al_2_O_3_ substrates (**a**); enlarged low-field part of the dependence (**b**). Arrows show the beginning of the measurement cycle starting from *B* = 0.

A permanent electromagnet was used to measure the resistance change of the films in a low magnetic field. The samples were first cooled in a zero magnetic field to 80 K, and then the magnetic field was increased to 0.7 T and decreased to zero. After that, the direction of the field was changed and the field increased again. Figure 4a shows the result of the resistivity change of the films grown on Si/SiO_2_ and Al_2_O_3_ substrates during several cycles of the magnetic-field change in the range of −0.7 T to +0.7 T. Figure 4b shows the enlarged portion of Figure 4a in the range of −0.15 T to +0.15 T. The arrows indicate the first cycle of the measurement starting from *B* = 0. After each cycle, the film resistance did not return to its initial value *ρ*_0_ and remained lower than before the measurement. This difference is called remnant resistivity and is marked by Δ*ρ*_remn_. To compare different films, the dependence of *ρ*(*B*) was normalized to the resistivity at the zero field *ρ*(0), as can be seen in Figure 4. The difference between the initial resistivity *ρ*_0_ and the resistivity *ρ*(0) is marked on the graph as Δ*ρ*_remn_.

After changing the magnetic-field direction, an increase in resistivity to a certain maximum was observed, followed by a sharp decrease at the low field and then a smoother dependence at higher fields. Such properties of the films in a magnetic field (positive magnetoresistance, followed by a sharp drop) are characteristics of polycrystalline manganite films consisting of crystallites separated by high-resistivity grain boundaries. The abrupt resistance change is known as low-field magnetoresistance (LFMR) and is explained by the spin-polarized tunneling effect [37,38]. Li et al. showed that the position of the resistivity peak matches the coercive magnetic field on the film-magnetization-hysteresis loop (when total magnetization equals zero) and corresponds to a state in which magnetic spins in individual domains incline to their respective easy axes, resulting in the largest angle between spins in neighboring domains [39]. In our case, the coercive magnetic field determined from these maxima was approx. 0.2 T for LSMO/Al_2_O_3_ and approx. 0.3 T for LSMO/SiO_2_/Si film. A higher magnetic field can align the spins to the field direction, drastically reducing the resistance.

Comparing the magnetoresistance behavior of the films deposited on different substrates, it can be concluded that the films on SiO_2_ showed lower remnant resistivity, lower LFMR values, and a peak of maximum resistivity at a higher magnetic field in comparison to the films deposited on Al_2_O_3_ substrate. More detailed investigations of remnant resistivity are presented in the next subsection using pulsed magnetic fields.

Despite the potential benefits of the grown manganite films, the presence of remnant resistivity and positive magnetoresistance is a drawback for cryogenic magnetic sensors, whose operation range should be extended up to several Tesla. However, at temperatures above the phase transition, the films are in a paramagnetic state and the remnant resistivity drops to zero after the magnetic field is removed; thus, the film’s resistance returns to its initial value.

**Figure 5:**
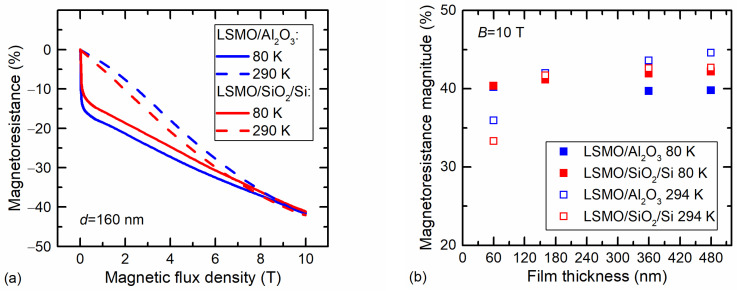
(**a**) Magnetoresistance dependence on magnetic-flux density at two ambient temperatures (80 K and 294 K) for 160 nm-thick LSMO/Al_2_O_3_ and LSMO/SiO_2_/Si films. (**b**) The *MR* magnitude for films with different thicknesses measured at 10 T.

The high-field magnetoresistance was measured in pulsed magnetic fields up to 10 T at two ambient temperatures. The *MR* dependences on magnetic-flux density are presented in Figure 5a. An abrupt *MR* change at a low field at 80 K was observed. This was a result of the low-field magnetoresistance, which is more evident in Figure 4.

Figure 5b presents the *MR* values at 10 T for films with different thicknesses. It can be seen that the *MR* values only slightly differed depending on the film substrate and ambient temperature. The smallest values were obtained for 60 nm films at room temperature, because the metal-insulator transition temperature of thin films was low enough in comparison with the measurement temperature (294 K) at which the films were in a paramagnetic state. The highest *MR* values at room temperature were obtained for the thicker films grown on Al_2_O_3_, which can be explained by the largest *T*_m_ values (see Table 1).

### 3.3. Resistance Relaxation of LSMO Films

**Figure 6:**
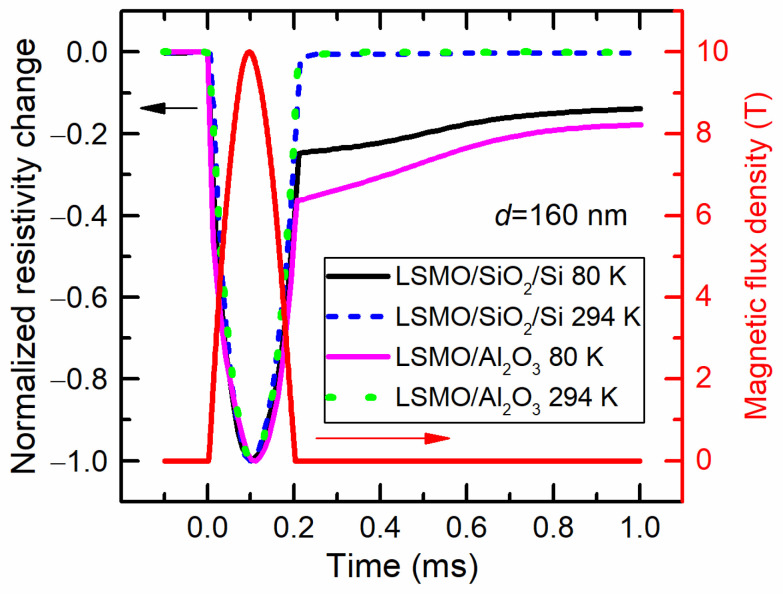
Normalized resistivity change of LSMO films with a thickness of 160 nm grown on Si/SiO_2_ and Al_2_O_3_ substrates (left scale) and magnetic-field pulse (right scale). Measurements performed at temperatures of 80 K and 294 K.

The resistance relaxation of LSMO films was investigated by using a single-pulsed magnetic field. Due to the design of a special coil with a non-metallic casing for the magnetic-pulse generator, the magnetic-field pulse had an abrupt switch-off, and it was possible to investigate the resistance-relaxation phenomena after removing the magnetic field. Figure 6 presents a typical magnetic-field pulse with an amplitude of 10 T and a duration of 200 μs (right scale) and normalized to the maximal resistivity change over time of two manganite films grown on different substrates (left scale). The measurements were performed at room (294 K) and cryogenic (80 K) temperatures.

Most of the film’s resistance dynamics followed the magnetic-field pulse. It is worth mentioning that due to the CMR effect, the resistance decreased with the increase in the magnetic field. However, after the abrupt switch-off of the magnetic-field pulse, the resistance relaxation was observed at a low temperature, whereas at room temperature it was diminished. Moreover, two relaxation mechanisms were distinguished, the first of which was much faster. For LSMO grown on SiO_2_ the remnant normalized resistivity was smaller.

**Figure 7:**
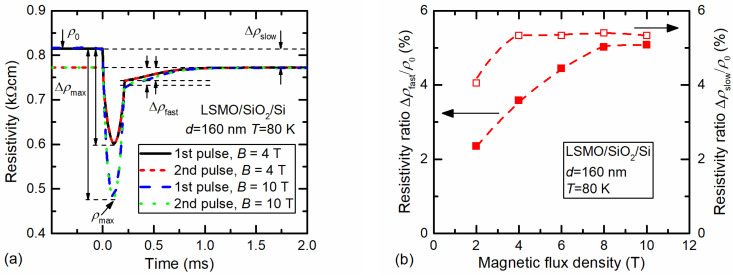
(**a**) Resistivity dependence over time of LSMO film with a thickness of 160 nm grown on Si/SiO_2_ substrate after the application of two single magnetic-field pulses of the same amplitude (4 T or 10 T) after cooling the sample down to 80 K. (**b**) Dependences of normalized resistivity of fast (solid symbols, left scale) and slow (open symbols, right scale) processes on magnetic-flux density.

Figure 7a presents the resistivity change over time for LSMO/SiO_2_/Si film after affecting it by two single pulses at 80 K.

The measurements were performed in the following way. After cooling the sample down to 80 K, the first magnetic-field pulse was applied. Figure 7a shows that the resistance relaxation after the magnetic field was switched off had two components: fast and slow. The fast component relaxed to a so-called slow resistivity value during several hundred microseconds. The observed slow relaxation in the measurement time scale (up to 10 ms) was constant. It is evident that at high magnetic fields (4–10 T) this slow remnant resistivity did not depend on magnetic-field amplitude. The second pulse was applied after a few minutes. Figure 7a demonstrates that zero-field resistivity in this case coincided with a slow remnant-resistivity value, which confirms that slow relaxation takes much more time. After that, the sample was warmed up to room temperature, then cooled down to 80 K again, and then the third pulse (not presented in Figure 7a) was applied. The resistivity change coincided well with the resistivity after the first pulse, starting from the same *ρ*_0_ value, which demonstrates that at room temperature the film returned to its initial paramagnetic state, as well as after cooling down again to 80 K to its zero-field ferromagnetic state without remnant resistivity, which was determined by film magnetization at this temperature.

The dependence of the remnant resistivity of fast and slow processes on magnetic-flux density for 160 nm-thick LSMO/SiO_2_/Si film is presented in Figure 7b. The normalized remnant fast resistivity increased with the magnetic field and tended to saturation approaching 10 T. The obtained results can be explained as follows. It is known that for good-quality epitaxial manganite films, the MR starts to saturate at ~10 T [40], which means that almost all magnetic domains are aligned by the application of magnetic field. For polycrystalline (nanostructured) films, the MR starts to saturate at much higher fields (B > 40 T, see [12,41]). Therefore, the amplitude of remnant resistivity is related to the magnetization and its relaxation in crystallites. The applied magnetic field aligns spins and the magnetization of the film increases, leading to increased remnant resistivity of the fast process.

The slow remnant resistivity in manganites is usually attributed to the interactions of magnetic moments in disordered systems, and their dynamics are considered using logarithmic law [18,19]. A more general model was suggested by Kohlrausch, and later Williams and Watts (KWW model) [23], describing the relaxation dynamics with a stretched exponent (~exp[−(*t*/*τ*_slow_) ^β^], where *τ*_slow_ is the characteristic time constant of the slow process 0 < β < 1). The KWW model usually considers disordered materials with spin-glass properties [21,24]. In our case, the defects in disordered grain boundaries (GBs) and broken bonds of the lattice of the crystallite surface behaved like pinning centers of magnetic flux, and relaxation to an equilibrium state was long enough at low temperatures. At magnetic fields higher than 2 T it was almost independent on the field amplitude because the coercive field of these films was 0.2–0.3 T. After the removal of magnetic fields of higher amplitudes, the film remained magnetized at a constant temperature (80 K) for a relatively long time. In our case, the slow process was almost constant in a time window of up to 10 ms. The goal of this study was to investigate manganite-film properties for fast-magnetic-sensor application. Our measurement setup did not allow for a stable temperature to be ensured for measurements for a much longer time; thus, the fitting results to the KWW model yielded a constant remnant resistivity of slow relaxation of up to 10 ms. For comparison, investigations of the slow relaxation of the polycrystalline manganite films were performed by other authors in time windows from 50 s to up to 10,000 s [18].

**Figure 8:**
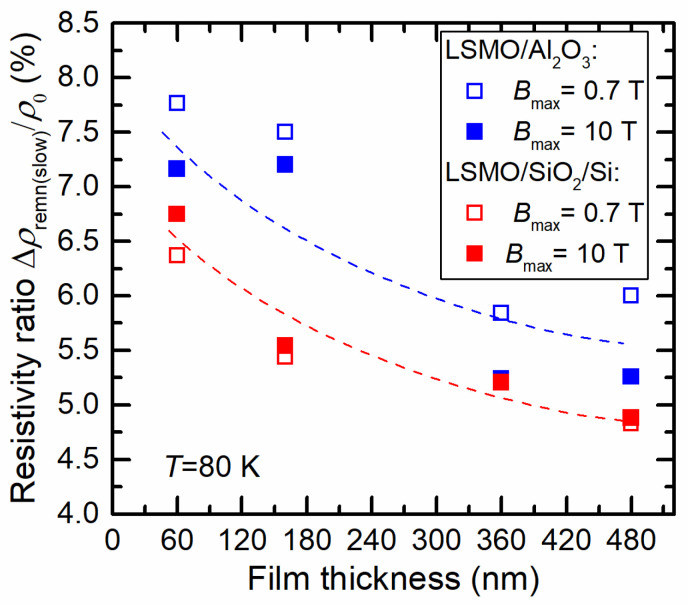
Normalized slow (remnant) resistivity dependence on film thickness for LSMO films grown on Si/SiO_2_ and Al_2_O_3_ substrates. Solid symbols represent measurements in a pulsed magnetic field of up to 10 T, open symbols show values obtained in a permanent magnetic field increasing up to 0.7 T and decreasing to 0 T.

Figure 8 presents a comparison of slow (remnant) resistivity on film thickness for two different substrates, with measurements performed in a permanent magnetic field of up to 0.7 T (see Figure 4) and a pulsed field of up to 10 T. For comparison of both films, the resistivity change was normalized to the initial zero-field resistivity *ρ*_0_. Figure 8 demonstrates that the normalized slow (remnant) resistivity values differed only slightly for both low- and high-field cases. This shows that the saturation magnetization of the films was achieved at relatively low magnetic fields. Furthermore, the normalized remnant resistivity decreased with the film thickness, which was a result of the better structural quality of thicker films having larger crystallites. The smaller slow (remnant) resistivity for LSMO films grown on Si/SiO_2_ substrate in comparison to LSMO/Al_2_O_3_ films can be explained by the interplay of defects acting as magnetic-flux pinning centers and the formation of more nucleation centers of twinned crystallites supporting the relaxation process.

**Figure 9:**
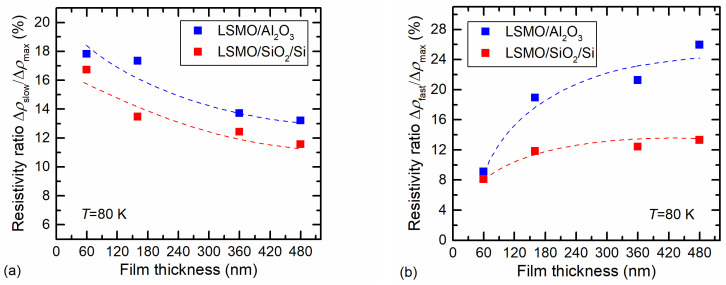
Dependence of the ratio of slow remnant resistivity (**a**) and fast remnant resistivity (**b**) with maximal-resistivity change during magnetic-field pulse vs. film thickness for LSMO films grown on Si/SiO_2_ and Al_2_O_3_ substrates.

It is evident that for sensor applications, the slow remnant resistivity is a disadvantage; thus, the Si/SiO_2_ substrate is favorable in comparison to Al_2_O_3_. If the measurements are performed at a constant cryogenic temperature and not changing the magnetic field direction to the opposite, the sensor can be calibrated by taking into account the remnant resistivity value, which almost does not depend on the magnetic-field amplitude. To increase the measurement accuracy, the magnetic sensor based on manganite film has to show high sensitivity (to demonstrate high magnetoresistance values) and a remnant resistivity that is as low as possible. Figure 9a presents the ratio of the slow remnant-resistivity change with its maximal change at the maximal magnetic field, i.e., Δ*ρ*_slow_/Δ*ρ*_max_ (the meaning of these resistivities is shown in Figure 7a). Figure 9a demonstrates that this ratio was lower for LSMO grown on Si/SiO_2_ substrate and for films with larger thicknesses.

**Figure 10:**
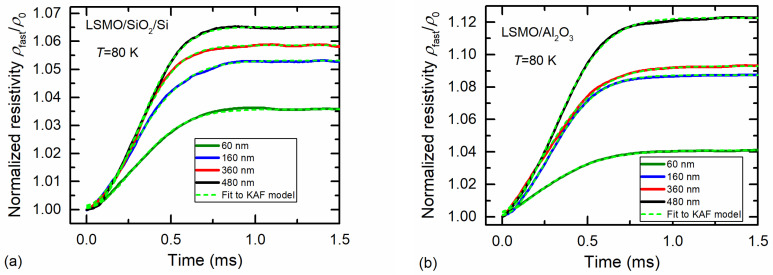
Fast remnant-resistivity dynamics normalized to the resistivity value at time instant *t* = 0, representing the time of the switch-off of the magnetic-field pulse: (**a**) for LSMO films grown on Si/SiO_2_ and (**b**) Al_2_O_3_ substrates. Light-green curves represent the results when fitted to the KAF model with the compressed exponent.

For measurement of short magnetic-field pulses (microsecond duration), the problem can occur due to so-called fast remnant resistivity, during which relaxation process takes place during several hundreds of microseconds. Figure 9b presents the ratio of the fast remnant resistivity with the maximal-resistivity change Δ*ρ*_fast_/Δ*ρ*_max_ (these resistivities are shown in Figure 7a). This ratio increased with the increase in film thickness and had a tendency toward saturation. This increase can be explained by the better structural quality and larger dimensions of the crystallites in thicker films, leading to higher magnetization and thus higher remnant resistivity of the fast process. As for the case of slow relaxation, lower values of the remnant resistivity of the fast process were observed for LSMO/SiO_2_/Si films, which were more disordered. Since the fast-relaxation dynamics exhibited an S-shaped form, they were studied using the Kolmogorov–Avrami–Fatuzzo (KAF) model [42,43,44], which considers the reorientation of magnetic domains towards an equilibrium state when the magnetic field is switched off. Therefore, the measured-resistivity *ρ* data of the fast process were recalculated to conductivity *σ* values and fitted to a compressed exponential decay [21]:

*σ*(*t*) = 1/*ρ*(*t*) = *σ*_0fast_ + Δ*σ*_fast_ exp[−(*t*/*τ*_fast_) ^β^], 1 < β < 3, (1)

where *σ*_fast_ is the remnant-conductivity amplitude, *σ*_0fast_ is the conductivity when the fast relaxation process is considered finished, and *τ*_fast_ is the time constant of the process. Figure 10 presents the dynamics of the remnant resistivity *ρ*_fast_(*t*) of the fast relaxation process (solid curves) normalized to the initial remnant resistivity *ρ*_fast_(0), where *t* = 0 is set at the time instant when the magnetic field is switched off. The remnant-resistivity magnitude was smaller for films grown on Si/SiO_2_ substrate and increased with the increase in film thickness. The dashed light-green curves represent fitting results to the Kolmogorov–Avrami–Fatuzzo model (see Equation (1)).

Figure 10 demonstrates that the compressed exponent described the fast relaxation process sufficiently well. The obtained values of the fitting parameter β were in the range of 1.8–2.1 for LSMO/SiO_2_/Si and 1.5–1.8 for LSMO/Al_2_O_3_ films, whereas *τ*_fast_ was in the range of 380–400 μs for LSMO/SiO_2_/Si and in the range of 360–400 μs for LSMO/Al_2_O_3_. It is worth mentioning that β values lower than 3 indicate that the relaxation to equilibrium state of the sample more likely took place through nucleation centers of many magnetic domains than through the growth of a few domains [21]. It has to be noted that the comparison of characteristic time constants of different films can be difficult when the parameter β varies. To address this issue, we evaluated the time *t*_1/2_ required for half of the film-remnant-resistivity magnitude to relax, as detailed in [45]. The *t*_1/2_ values obtained were the following: 315–330 μs for LSMO/SiO_2_/Si and 308–330 μs for LSMO/Al_2_O_3_ films. The smallest *t*_1/2_ and the smallest fast remnant resistivity were obtained for 160 nm-thick films. Therefore, these films could be used for the development of fast magnetic sensors operating at cryogenic temperatures.

### 3.4. Testing of Magnetic-Field Sensor Based on LSMO/SiO_2_/Si Film

**Figure 11:**
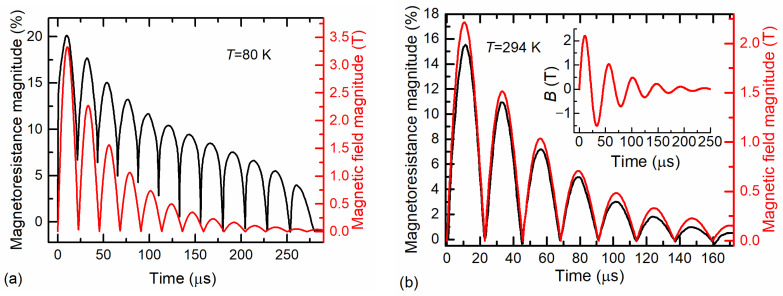
Magnetoresistance dynamics (black curves) of 160 nm-thick LSMO/SiO_2_/Si film when an alternative magnetic field with damping amplitude was applied. The measurements were performed at ambient temperature of 80 K (**a**) and 294 K (**b**). The red curves represent the time-dependant magnitude of the applied magnetic field. The inset shows the real magnetic-field waveform.

The LSMO/SiO_2_/Si film with a thickness of 160 nm was used for the fabrication of magnetic-field sensor with two electrodes and bifilarly twisted wires for signal transmission (see, for example, [32]). The testing of the sensor was performed by using a generator that produced an alternating magnetic field with a half-period of approximately 22 µs and dumped amplitude. Figure 11a,b illustrates the magnetoresistance dynamics of the sensor at 80 K and room temperature (294 K), respectively. The alternating magnetoresistance over the time was observed as an alternating magnetic field (see inset in Figure 11b). It is worth mentioning that the sensor’s magnetoresistance was determined only by the magnitude of the magnetic field, not its orientation in respect to the film plane. For signal comparison, the magnitude of the applied alternating magnetic field measured by a pick-up coil is shown in the main graphs of Figure 11a,b.

Figure 11a shows the sensor’s magnetoresistance variation in time at 80 K during the applied alternating magnetic field. At the beginning of the applied field, an abrupt change in the magnetoresistance of the sensor was observed. This was caused by the so-called low-field magnetoresistance effect, which can clearly be seen in Figure 4. After reaching the maximum of the field, the *MR* of the sensor started to decrease, following the magnetic-field waveform; however, at *B* = 0 the *MR* did not decrease to zero. This result demonstrates that the LSMO film at 80 K had remnant resistivity due to the magnetic-memory effects observed when a single magnetic-field pulse was applied (see Figure 6 and Figure 7). Moreover, Figure 11a shows that when the magnetic field changed its direction (after each half-period) and the full demagnetization of the films should have been achieved already at the coercive magnetic field of ~0.3 T, the resistance of the film remained lower in comparison with the initial resistance before the magnetic field was applied. The following oscillation of the magnetic field with decreasing amplitude led to a drop in magnetoresistance to zero after a number of pulses. This means that the alternating magnetic field of both polarities with decreasing amplitude demagnetized the film, and its resistance increased to the initial value.

The testing results of the LSMO/SiO_2_/Si-based sensors in an alternating magnetic field at room temperature are shown in Figure 11b. In this case, the change in the magnetoresistance of the films followed the change in the magnetic-field magnitude well. At the same time, no memory effects were observed, and the magnetoresistance at zero magnetic fields was equal to zero. Therefore, the LSMO films grown on the Si/SiO_2_ substrate are suitable for the development of magnetic-field sensors capable of measuring short alternating magnetic fields at room temperature.

## 4. Conclusions

In conclusion, the investigation of magnetoresistance and its relaxation in La_0.83_Sr_0.17_Mn_1.21_O_3_ (LSMO) films under pulsed magnetic fields revealed the possibility of employing Si/SiO_2_ substrate for depositing nanostructured films and achieving the key parameters required for magnetic-sensor applications.

It was found that the film thickness determines the microstructure of the LSMO films, resulting in the smallest crystallites (average dimensions of ~40 nm) in 60 nm-thick films in comparison to the larger crystallites (~200 nm) in 480 nm films. It was seen that the metal-insulator transition temperature *T*_m_ increased and the resistivity maximum *ρ*_m_ corresponding to this temperature decreased with the increase in film thickness up to 360 nm, which was a result of the decrease in the amount of disordered grain-boundary material with the increase in crystallite size. The Si/SiO_2_ substrate with an amorphous surface resulted in higher resistivity values of the LSMO films in comparison with the films grown on polycrystalline Al_2_O_3_ substrate.

The magnetoresistance values obtained upon application of a pulsed magnetic field with an amplitude of 10 T were comparable for all investigated samples (≈−40%) at cryogenic and room temperatures.

The resistance-relaxation dynamics investigated after the magnetic-field pulse was switched off revealed two components: fast, occurring over a time scale of several hundred microseconds, and slow, which remained constant during the time window of measurements up to 10 ms. It was found that the fast relaxation process could be analyzed using the Kolmogorov–Avrami–Fatuzzo model utilizing a compressed exponential expression. The values of the exponent parameter, denoted as β, were found to range from 1.5 to 2.1, which indicates that the predominant mechanism responsible for this relaxation process was the nucleation of magnetic domains in crystallites. It was determined that the remnant resistivity was smaller for the LSMO films grown on Si/SiO_2_ substrate in comparison to the LSMO/Al_2_O_3_ films.

The testing of the LSMO/SiO_2_/Si-based magnetic sensors in alternating magnetic fields with a half-period of 22 μs demonstrated that these films can be used for the development of fast magnetic sensors operating at room temperature. For operation at cryogenic temperature, the LSMO/SiO_2_/Si films can be employed only for single-pulse measurements due to magnetic-memory effects.

## Figures and Tables

**Table 1:** *T*_m_ and *ρ*_m_ values for LSMO films with different thicknesses grown on different substrates: Si/SiO_2_ and Al_2_O_3_.

Substrate	Film Thickness, nm	*ρ*_m_, Ω cm	*T*_m_, K
Si/SiO_2_	60	2.79	240
	160	1.36	250
	360	1.06	260
	480	1.26	257
Al_2_O_3_	60	1.92	248
	160	0.93	260
	360	0.44	285
	480	0.68	272

## Data Availability

Not applicable.

## References

[1] Jogschies L., Klaas D., Kruppe R., Rittinger J., Taptimthong P., Wienecke A., Rissing L., Wurz M.C. (2015). Recent developments of magnetoresistive sensors for industrial applications. Sensors.

[2] Žurauskienė N. (2023). Engineering of Advanced Materials for High Magnetic Field Sensing: A Review. Sensors.

[3] Yole Intelligence (2022). Magnetic Sensor 2022 Report.

[4] Zheng C., Zhu K., Cardoso de Freitas S., Chang J.-Y., Davies J.E., Eames P., Freitas P.P., Kazakova O., Kim C.G., Leung C.-W. (2019). Magnetoresistive sensor development roadmap (non-recording applications). IEEE Trans. Magn..

[5] Battesti R., Beard J., Böser S., Bruyant N., Budker D., Crooker S.A., Daw E.J., Flambaum V.V., Inada T., Irastorza I.G. (2018). High Magnetic Fields for Fundamental Physics. Phys. Rep..

[6] Coey J.M.D., Viret M., von Molnár S. (2009). Mixed-Valence Manganites. Adv. Phys..

[7] Zhao B., Hu X., Dong F., Wang Y., Wang H., Tan W., Huo D. (2022). The Magnetic Properties and Magnetocaloric Effect of Pr_0.7_Sr_0.3_MnO_3_ Thin Film Grown on SrTiO_3_ Substrate. Materials.

[8] Israel C., Calderón M.J., Mathur N.D. (2007). The Current Spin on Manganites. Mater. Today.

[9] Dong C., Chen R., Liu Y., Liu C., Zhu H., Ke J., Liu W., Yang M., Wang J. (2019). Field-Induced Magnetic Phase Transitions and Rich Phase Diagram of HoMnO_3_ Single Crystal. Crystals.

[10] Millis A.J., Littlewood P.B., Shraiman B.I. (1995). Double Exchange Alone Does Not Explain the Resistivity of La_1−X_Sr_x_MnO_3_. Phys. Rev. Lett..

[11] Ziese M. (2002). Extrinsic Magnetotransport Phenomena in Ferromagnetic Oxides. Rep. Prog. Phys..

[12] Pękała M., Pękała K., Drozd V. (2015). Magnetotransport Study of Nanocrystalline and Polycrystalline Manganites La_0.8_Sr_0.2_MnO_3_ in High Magnetic Fields. J. Appl. Phys..

[13] Zurauskiene N., Balevicius S., Stankevic V., Kersulis S., Schneider M., Liebfried O., Plausinaitiene V., Abrutis A. (2011). B-Scalar Sensor Using CMR Effect in Thin Polycrystalline Manganite Films. IEEE Trans. Plasma Sci..

[14] Balevičius S., Žurauskienė N., Stankevič V., Keršulis S., Plaušinaitienė V., Abrutis A., Zherlitsyn S., Herrmannsdörfer T., Wosnitza J., Wolff-Fabris F. (2012). Nanostructured Thin Manganite Films in Megagauss Magnetic Field. Appl. Phys. Lett..

[15] Stankevič T., Medišauskas L., Stankevič V., Balevičius S., Žurauskienė N., Liebfried O., Schneider M. (2014). Pulsed Magnetic Field Measurement System Based on Colossal Magnetoresistance-B-Scalar Sensors for Railgun Investigation. Rev. Sci. Instrum..

[16] Haran T.L., Hoffman R.B., Lane S.E. (2013). Diagnostic capabilities for electromagnetic railguns. IEEE Trans. Plasma Sci..

[17] Bellmann J., Lueg-Althoff J., Schulze S., Gies S., Beyer E., Erman Tekkaya A. (2016). Measurement and Analysis Technologies for Magnetic Pulse Welding: Established Methods and New Strategies. Adv. Manuf..

[18] Sirena M., Steren L.B., Guimpel J. (2001). Magnetic Relaxation in Bulk and Film Manganite Compounds. Phys. Rev. B.

[19] Kozlova N., Dörr K., Eckert D., Handstein A., Skourski Y., Walter T., Müller K.-H., Schultz L. (2003). Slow Relaxation of Grain Boundary Resistance in a Ferromagnetic Manganite. J. Appl. Phys..

[20] Žurauskienė N., Rudokas V., Keršulis S., Stankevič V., Pavilonis D., Plaušinaitienė V., Vagner M., Balevičius S. (2021). Magnetoresistance and Its Relaxation of Nanostructured La-Sr-Mn-Co-O Films: Application for Low Temperature Magnetic Sensors. J. Magn. Magn. Mater..

[21] Xi H., Gao K.-Z., Ouyang J., Shi Y., Yang Y. (2008). Slow Magnetization Relaxation and Reversal in Magnetic Thin Films. J. Phys. Condens. Matter.

[22] Bakaul S.R., Miao B.F., Lin W., Hu W., David A., Ding H.F., Wu T. (2012). Domain-Related Origin of Magnetic Relaxation in Compressively Strained Manganite Thin Films. Appl. Phys. Lett..

[23] Williams G., Watts D.C. (1970). Non-Symmetrical Dielectric Relaxation Behaviour Arising from a Simple Empirical Decay Function. Trans. Faraday Soc..

[24] Macdonald J.R., Phillips J.C. (2005). Topological Derivation of Shape Exponents for Stretched Exponential Relaxation. J. Chem. Phys..

[25] Costa T., Cardoso F.A., Germano J., Freitas P.P., Piedade M.S. (2017). A CMOS Front-End with Integrated Magnetoresistive Sensors for Biomolecular Recognition Detection Applications. IEEE Trans. Biomed. Circuits Syst..

[26] Khan M.A., Sun J., Li B., Przybysz A., Kosel J. (2021). Magnetic sensors-A review and recent technologies. Eng. Res. Express.

[27] Camacho R., Sánchez Llamazares J.L., Curiel M., Sánchez Llamazares J.L., Siqueiros J.M., Raymond Herrera O. (2020). Superparamagnetic State in La_0.7_Sr_0.3_MnO_3_ Thin Films Obtained by Rf-Sputtering. Sci. Rep..

[28] Shim I.-B., Kim C.-S., Park K.-T., Oh Y.-J. (2001). Role of intermediate layer for La_2/3_Sr_1/3_MnO_3_/SiO_2_/Si(1 0 0) granular thin films. J. Magn. Magn. Mater..

[29] Kang Y.-M., Ulyanov A.N., Lee S.-Y., Yoo S.-I. (2011). Low field magnetoresistance properties of (La_0.75_Sr_0.25_)_1.05_Mn_0.95_O_3_ polycrystalline thin films on a-SiO_2_/Si substrates prepared by ex-situ solid phase crystallization. Met. Mater. Int..

[30] Zurauskiene N., Stankevic V., Kersulis S., Vagner M., Plausinaitiene V., Dobilas J., Vasiliauskas R., Skapas M., Koliada M., Pietosa J. (2022). Enhancement of Room-Temperature Low-Field Magnetoresistance in Nanostructured Lanthanum Manganite Films for Magnetic Sensor Applications. Sensors.

[31] Lukose R., Plausinaitiene V., Vagner M., Zurauskiene N., Kersulis S., Kubilius V., Motiejuitis K., Knasiene B., Stankevic V., Saltyte Z. (2019). Relation between Thickness, Crystallite Size and Magnetoresistance of Nanostructured La_1−x_Sr_x_Mn_y_O_3±δ_ Films for Magnetic Field Sensors. Beilstein J. Nanotechnol..

[32] Stankevič V., Keršulis S., Dilys J., Bleizgys V., Viliūnas M., Vertelis V., Maneikis A., Rudokas V., Plaušinaitienė V., Žurauskienė N. (2023). Measurement System for Short-Pulsed Magnetic Fields. Sensors.

[33] Zurauskiene N., Balevicius S., Stankevic V., Kersulis S., Klimantavicius J., Plausinaitiene V., Kubilius V., Skapas M., Juskenas R., Navickas R. (2018). Magnetoresistive Properties of Thin Nanostructured Manganite Films Grown by Metalorganic Chemical Vapour Deposition onto Glass-Ceramics Substrates. J. Mater. Sci..

[34] Stankevic V., Zurauskiene N., Kersulis S., Plausinaitiene V., Lukose R., Klimantavicius J., Tolvaišienė S., Skapas M., Selskis A., Balevicius S. (2022). Nanostructured Manganite Films Grown by Pulsed Injection MOCVD: Tuning Low- and High-Field Magnetoresistive Properties for Sensors Applications. Sensors.

[35] Grainys A., Novickij J., Stankevič T., Stankevič V., Novickij V., Žurauskienė N. (2015). Single Pulse Calibration of Magnetic Field Sensors Using Mobile 43 KJ Facility. Meas. Sci. Rev..

[36] Zurauskiene N., Pavilonis D., Balevicius S., Stankevic V., Maneikis A., Plausinaitiene V., Novickij J. (2015). Fast Resistance Relaxation in Nanostructured La–Ca–Mn–O Films in Pulsed Magnetic Fields. IEEE Trans. Plasma Sci..

[37] Hwang H.Y., Cheong S.-W., Ong N.P., Batlogg B. (1996). Spin-Polarized Intergrain Tunneling inLa_2/3_Sr_1/3_MnO_3_. Phys. Rev. Lett..

[38] Lee S., Hwang H.Y., Shraiman B.I., Ratcliff W.D., Cheong S.-W. (1999). Intergrain magnetoresistance via second-order tunneling in perovskite manganites. Phys. Rev. Lett..

[39] Li J., Wang P., Xiang J.Y., Zhu X.H., Peng W., Chen Y.F., Zheng D.N., Li Z.W. (2005). Large Low-Field Magnetoresistance Observed in Twinned La_2∕3_Ca_1∕3_MnO_3_ Thin Films Epitaxially Grown on Yttria-Stabilized Zirconia-Buffered Silicon on Insulator Substrates. Appl. Phys. Lett..

[40] Wagner P., Gordon I., Trappeniers L., Vanacken J., Herlach F., Moshchalkov V.V., Bruynseraede Y. (1998). Spin Dependent Hopping and Colossal Negative Magnetoresistance in EpitaxialNd_0.52_Sr_0.48_MnO_3_ Films in Fields up to 50 T. Phys. Rev. Lett..

[41] Zurauskiene N., Balevicius S., Pavilonis D., Stankevic V., Plausinaitiene V., Zherlitsyn S., Herrmannsdorfer T., Law J.M., Wosnitza J. (2014). Magnetoresistance and Resistance Relaxation of Nanostructured La-Ca-MnO Films in Pulsed Magnetic Fields. IEEE Trans. Magn..

[42] Kolmogorov A. (1937). On the Statistical Theory of Metal Crystallization. Izv. Akad. Nauk. SSSR Ser. Mat..

[43] Avrami M. (1940). Kinetics of Phase Change. II Transformation-time Relations for Random Distribution of Nuclei. J. Chem. Phys..

[44] Fatuzzo E. (1962). Theoretical Considerations on the Switching Transient in Ferroelectrics. Phys. Rev..

[45] Zurauskiene N., Juskenas R., Pavilonis D., Klimantavicius J., Balevicius S., Stankevic V., Vasiliauskas R., Plausinaitiene V., Abrutis A., Skapas M. (2017). Magnetoresistance Relaxation Anisotropy of Nanostructured La-Sr(Ca)-Mn-O Films Induced by High-Pulsed Magnetic Fields. IEEE Trans. Plasma Sci..

